# Intercellular Communication Pathways in Cerebral Ischemia: Mechanisms, Molecular Insights, and Therapeutic Implications

**DOI:** 10.2174/011570159X388985250711064325

**Published:** 2025-07-18

**Authors:** Guoqian Cui, Wenbo Guo, Muzi Li, Shengshuang Chen, Xin Shao, Jie Liao, Xiaohui Fan

**Affiliations:** 1 College of Pharmaceutical Engineering of Traditional Chinese Medicine, Tianjin University of Traditional Chinese Medicine, Tianjin, 300193, China;; 2 College of Pharmaceutical Sciences, Zhejiang University, Hangzhou, 310058, China;; 3 Zhejiang Key Laboratory of Chinese Medicine Modernization, Innovation Center of Yangtze River Delta, Zhejiang University, Jiaxing, 314100, China;; 4 The Joint-Laboratory of Clinical Multi-omics Research between Zhejiang University and Ningbo Municipal Hospital of TCM, Ningbo Municipal Hospital of TCM, Ningbo, 315100, China

**Keywords:** Cerebral ischemia, intercellular communication, inflammatory response, neuroprotection, regenerative mechanisms, injury

## Abstract

**Introduction:**

Cerebral ischemia (CI) is a severe neurological disorder characterized by high incidence and disability rates. Its pathogenesis is complex, involving multiple interrelated biological processes. Among these, intercellular communication has emerged as a key mechanism regulating the damage and recovery phases of CI. It controls information exchange between cells, thereby playing a crucial role in cellular responses to ischemic injury. Understanding how intercellular communication promotes the pathophysiology of CI may provide valuable insights into new therapeutic targets.

**Methods:**

To elucidate the role of intercellular communication in CI, recent literature was analyzed, with a focus on how intercellular communication influences cellular behaviors and metabolism. This review integrates data from molecular biology, cellular signaling studies, and cerebral ischemia models.

**Results:**

Studies indicate that intercellular communication significantly influences the progression and outcomes of CI. Intercellular communication not only participates in regulating the inflammatory response following injury but also plays a dual role in neuroprotection and regeneration.

**Discussion:**

The dual role of intercellular communication—exacerbating damage through inflammatory cascades and promoting recovery through neuroprotective mechanisms—highlights its complex contribution to the pathology of CI. Cellular crosstalk between neurons, glial cells, endothelial cells, and immune cells coordinates the dynamic response to ischemic injury. Understanding these dynamics offers promising opportunities for targeted interventions.

**Conclusion:**

Intercellular communication plays a central role in the mechanisms of injury and repair in cerebral ischemia. By influencing inflammation, neuroprotection, and regeneration, it serves as both a mediator of injury and a potential therapeutic target. Further research is needed to fully elucidate these mechanisms and translate them into effective clinical strategies for treating CI.

## 1 INTRODUCTION

Cerebral Ischemia (CI) is a serious disease caused by insufficient blood supply to the brain, and its complex pathomechanism involves the interaction of multiple cell types, such as neurons, glial cells, and endothelial cells [1]. During the onset of CI, intercellular communication has a profound impact, which not only affects cell survival and death, but also plays a key role in tissue repair and regeneration [2]. In the early stage of ischemia, injury signals trigger a complex network of intercellular communication, including a series of processes, such as inflammatory response, neuroprotection, and repair. Intercellular signaling can be achieved through a variety of mechanisms, including cell contact, lysis factors, and extracellular vesicles. These communication mechanisms enable the dynamic regulation of cell survival, functional regulation, and tissue repair through various pathways, including the secretion of cytokines, the release of exosomes, and direct cell contact [3]. The complexity of these mechanisms makes intercellular communication a major area of research.

 In recent years, scientists have greatly improved their understanding of intercellular communication. Studies have shown that intercellular interactions not only regulate cell differentiation and function, but are also indispensable in the occurrence and development of various diseases, such as neurodegenerative diseases, cardiovascular diseases, and metabolic diseases [4]. For example, in CI, intercellular signaling can lead to neuronal damage and death [5]. Communication pathways, such as exosomes and nanotubes, play a key role in regulating neuroinflammation and promoting vascular remodeling and neuroregeneration [6]. The dynamics of these mechanisms under pathological conditions and their specific therapeutic potential are not fully understood. Meanwhile, the development of technologies, such as single-cell RNA sequencing technology(scRNA-seq) and spatial omics, provides unprecedented opportunities to unravel the complex communication networks between cells. The rise of spatial transcriptomics and other spatial omics techniques provides significant support for a deeper understanding of the communication modes of various cells in specific microenvironments during CI.

 This review systematically searched the following databases: PubMed, Web of Science, Embase, *etc*. The keywords used for retrieval included: “Cerebral ischemia”, “Intercellular communication”, “Single cell sequencing”, “Neuroinflammation", “Stroke”, “Herbal medicine”, *etc*., and were combined with Boolean logic words (AND, OR) for retrieval. Inclusion criteria were based on the following conditions: ① Animal experiments or mechanism studies related to the treatment of cerebral ischemia/reperfusion injury; ② Original research or systematic review; and ③ Public literature published in English or Chinese. A qualitative analysis of literature that met the inclusion criteria was conducted, and the results were organized and classified based on core themes.

**Fig. (1):**
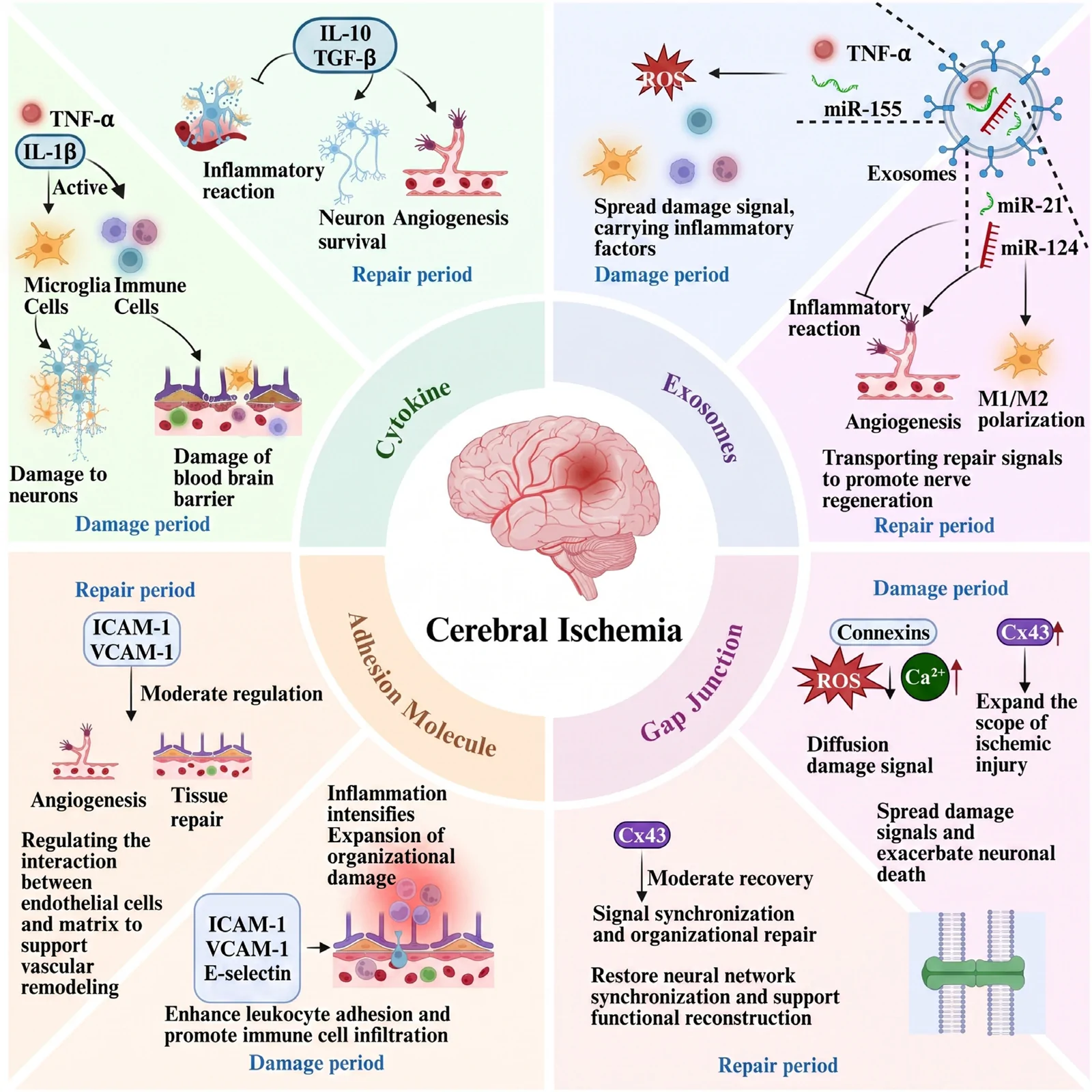
Major intercellular communication mechanisms in CI.

This paper reviews the major intercellular communication pathways in CI, including cytokines, exosomes, gap junctions, and adhesion molecules, systematically summarizing their dual roles in cerebral ischemic injury and repair (Fig. **1**). The limitations of related studies and future research directions are discussed, aiming to provide a theoretical basis and new ideas for cell communication-targeted therapy.

## 2 GLIAL-NEURONAL INTERACTIONS IN CI

Glial cells play a complex role in CI, and they are not only involved in the early damage response of neurons, but also have a key supporting role in the subsequent repair process [7, 8]. In the early stages of CI, glial cells recruit immune cells to the site of injury by releasing cytokines and chemokines, thereby triggering an inflammatory response that clears the injured cells but may also lead to further damage to neighboring healthy cells [9, 10]. However, during the recovery phase after ischemia, glial cells can, in turn, promote neuronal survival and regeneration through the secretion of neurotrophic factors, such as brain-derived neurotrophic factor (BDNF) and nerve growth factor (NGF), thereby demonstrating their dual role in CI [11, 12].

### 2.1 The Role of Glial Cells and Neurons in CI: Early Damage Response and Late Repair

 After CI, the early response of glial cells is to cope with the injury caused by ischemia through cell activation and morphological changes. The interactions between glial cells and neurons after CI are classified and elaborated based on the types of glial cells.

Microglia are the primary immune cells in the central nervous system, responsible for monitoring and clearing damaged cells. In the case of CI, the activation of microglia has a complex dual role, which can not only provide neuroprotection, but also lead to nerve injury. Studies have shown that microglia are rapidly activated after ischemia, releasing a variety of cytokines and chemokines that, to some extent, can promote the inflammatory response and attract additional immune cells to the damaged area, thereby exacerbating the injury [13]. However, on the other hand, microglia can also promote nerve repair by clearing apoptotic neurons and releasing neurotrophic factors. For example, M2 microglia have been found to inhibit glial scar formation by secreting small exosomes and miRNAs, such as miR-124, and enhance the recovery ability after CI by promoting neuronal recovery and plasticity [14].

Astrocytes play a crucial role in maintaining the integrity of the blood-brain barrier (BBB) and regulating the neuronal microenvironment. When CI occurs, the reactivity of astrocytes is significantly enhanced, as evidenced by an increase in cell volume and the expansion of cell processes. This reactivity may lead to the protection or damage of neurons. Studies have found that astrocytes release a variety of inflammatory factors and cytokines after CI, which can not only promote neuronal survival, but also inhibit nerve regeneration by producing glial scar [15]. For example, astrocytes promote neuronal survival and functional recovery by secreting neurotrophic factors, such as glial cell-derived neurotrophic factor [16]. However, excessive reactivity after CI may also lead to neuronal damage and dysfunction.

Oligodendrocytes are responsible for the formation and maintenance of myelin sheaths in the central nervous system. CI events can lead to the dysfunction of oligodendrocytes and then affect the transmission of neural signals. Studies have shown that the death and functional decline of oligodendrocytes following CI can significantly impact the myelination of neurons, leading to delayed signal transmission and loss of neural function [17]. In addition, oligodendrocytes play an important role in the repair process after CI. Their regeneration ability and myelination ability are directly related to the recovery of neurons and the reconstruction of brain function. Studies have found that defects in oligodendrocytes are associated with various neurodegenerative diseases, and understanding their survival and regeneration mechanisms after CI is crucial for the development of new therapies [18].

### 2.2 Signaling between Glial Cells and Neurons: Synergy through Molecules, such as Cytokines and Neurotransmitters

 The signal transmission processes between glial cells and neurons rely on various mechanisms, including the interaction of cytokines and neurotransmitters [19]. Astrocytes have a “sentinel” function that can sense changes in blood-borne molecules, thereby affecting neuronal signal conduction and the functional state of neurons [20]. Additionally, glial cells regulate neuronal excitability by releasing neurotransmitters, thereby helping to maintain neural network stability [21]. In specific contexts, glial cells can even modify how neurons adapt, such as participating in the process of olfactory adaptation. This suggests they play a much bigger role in information processing than just being "support cells" [22].

Glial cells are also involved in the regulation of neuronal growth and regeneration through signal transduction pathways [23]. For example, the Ras homologous gene family member A (RhoA) signaling pathway has been shown to be highly expressed in neurons and glial cells, playing a primary role in a wide range of cellular functions [24]. Abnormal signaling by RhoA has been associated with a wide range of neurodegenerative diseases, suggesting that regulating this signaling pathway may provide new ideas for the treatment of neurological injuries [24].

### 2.3 Communication Mechanism between Glial Cells, Vascular Cells, and Immune Cells

Glial cells not only provide support and nutrition in the central nervous system, but also maintain the health and function of the nervous system through complex communication mechanisms with neurons, vascular cells, and immune cells, especially in pathological conditions, such as CI.

Glial cells communicate in the central nervous system through various mechanisms, including cytokine and chemokine-mediated signal transmission, as well as direct cell-cell connections [25]. These communications not only regulate the activity of glial cells themselves, but also affect neuronal function and the stability of the blood-brain barrier. In pathological conditions such as CI, the expression of cytokines and chemokines is upregulated, promoting glial cell activation, an inflammatory response, and the repair process [26]. At the same time, gap junctions and synaptic associations ensure the rapid transmission and functional coordination of intercellular signals, which is essential for the homeostatic maintenance of the nervous system and self-repair after injury [27].

The interaction between glial cells and vascular cells plays a key role in maintaining the health of the central nervous system and disease progression. Astrocytes and microglia not only support neuronal function, but also affect the integrity of the blood-brain barrier by regulating the neurovascular unit, especially in pathological conditions, such as CI [28]. Glial cells promote vascular remodeling, regulate blood flow and vascular permeability, and affect immune cell infiltration and inflammatory response by secreting vascular endothelial growth factor (VEGF) [29]. At the same time, vascular endothelial cells undergo changes in function and morphology in response to glial cell-secreted factors, thereby affecting the stability of the blood-brain barrier [30]. Glial cells can not only protect the nervous system by enhancing the blood-brain barrier, but also promote its destruction and aggravate nerve injury under pathological conditions.

The interaction between glial cells and immune cells plays a key role in the health and disease of the central nervous system. Microglia, as the main resident immune cells, can sense pathological changes and regulate neuroinflammatory responses. For example, after ischemic brain injury, microglia affect the survival and repair of neural cells by releasing inflammatory mediators [31]. Microglia release pro-inflammatory factors in the inflammatory state, attracting peripheral immune cells such as T cells and macrophages to migrate to the center, forming a complex immune environment, which in turn affects disease progression [32]. After ischemia, the infiltration of immune cells can not only promote tissue repair, but also lead to secondary nerve injury due to excessive inflammatory reaction, especially M1 macrophages [33]. Glial cells not only act as executors of the immune response but also participate in neuroprotection and repair by regulating the permeability of the blood-brain barrier, releasing neurotrophic factors and cytokines, and affecting the migration and function of peripheral immune cells [34]. These complex interactions suggest that glial cells and immune cells have indispensable dual roles in the occurrence and development of neurological diseases. The interactions and contributions between astrocytes, microglia, oligodendrocytes, neurons, vascular cells, and immune cells after CI are summarized in Table **1**.

## 3 POST-ISCHEMIC ADHESION MOLECULE-MEDIATED CELLULAR COORDINATION

Following an ischemic event, intercellular adhesion molecules play a crucial role in regulating intercellular interactions and coordinating cellular responses. These molecules are able to mediate interactions between cellular coordination, which in turn affects neuronal cell survival, migration, and repair processes [35]. Following ischemia, endothelial cell activation leads to the increased expression of adhesion molecules, such as vascular cell adhesion molecule-1 (VCAM-1) and intercellular adhesion molecule-1 (ICAM-1), which promotes leukocyte recruitment and migration, thereby exacerbating inflammatory responses and tissue damage [36, 37]. Therefore, an in-depth understanding of the function of these adhesion molecules and their role in cellular coordination after ischemia is of great significance for the development of new therapeutic approaches.

### 3.1 The Role of Adhesion Molecules in CI

Adhesion molecules play a role in the pathophysiological process of CI, particularly in cell-cell interactions and signal transduction. Integrins and calmodulin are two major classes of adhesion molecules that play a crucial role in neuronal survival and functional recovery after CI by facilitating cell-cell and cell-matrix interactions [38].

During ischemia-reperfusion, the activation of integrins promotes endothelial cell proliferation and migration and improves vascular regeneration. Studies have shown that integrins αvβ3 and α5β1 promote angiogenesis and neuroprotection by mediating cell adhesion and migration during the repair of post-ischemic brain tissue [39]. Integrin-mediated signaling pathways can promote neovascularization by enhancing the expression of VEGF and hypoxia-inducible factor-1 alpha (HIF-1α) [40]. Meanwhile, integrins are also involved in regulating inflammatory responses, and after ischemia, the enhanced expression of integrins promotes the recruitment of inflammatory cells, which also exacerbates brain injury [41].

Equally important is the role of calcineurin after ischemia. Changes in the expression and function of cadherin are closely related to the permeability of the BBB, and changes in the expression of calcineurin after ischemia may lead to disruption of the BBB, which may exacerbate brain injury [42]. Excessive VEGF release leads to down-regulation of calcineurin expression and increased endothelial cell permeability, resulting in cerebral oedema and further neurological damage [43].

### 3.2 Cell Adhesion Regulates the Initial Response to Nerve Injury and the Coordination between Cells

 Cell adhesion plays a crucial role in the initial response after nerve injury, especially in the context of CI and reperfusion injury [44]. The neural damage caused by CI not only involves direct damage to neurons, but also accompanies dysfunction of microvessels and activation of inflammatory responses [45]. During this process, cell adhesion molecules affect the progression and repair of nerve damage by regulating intercellular interactions and signal transduction [46].

After CI, cell adhesion molecules play a central regulatory role in initiating inflammatory responses, neuronal glial cell interactions, and microenvironment remodeling [47]. In the early stage of CI, endothelial cells are activated, significantly upregulating the expression of adhesion molecules such as VCAM-1 and ICAM-1, promoting the recruitment and infiltration of immune cells, especially white blood cells, and triggering local inflammatory responses [48]. However, excessive inflammation can exacerbate nerve damage and lead to functional impairment. At the same time, the interaction between neurons and glial cells is also regulated by cell adhesion molecules [49]. The signals released by neurons after CI can activate astrocytes, inducing their expression of related adhesion molecules and enhancing adhesion and signal communication between the two, thereby promoting neuronal survival and functional recovery [50]. Among them, the protective effect of L1 cell adhesion molecules(L1CAM) is particularly significant, and their blockade can exacerbate nerve damage [51]. In addition, adhesion molecules also affect the neural microenvironment by regulating the permeability of the BBB, which can lead to barrier disruption during ischemia-reperfusion, allowing inflammatory cells and harmful substances to enter brain tissue and further aggravate damage [52]. Therefore, regulating the expression and function of cell adhesion molecules not only helps to limit inflammatory responses, but may also become a potential strategy for improving neurological prognosis [53]. At the same time, cell adhesion molecules also cooperate with signals, such as cytokines and growth factors, to promote neuronal migration, proliferation, and regeneration, playing a coordinating and supportive role throughout the entire repair process [54].

### 3.3 Relationship of Adhesion Molecules to Immune Response and Nerve Repair

Adhesion molecules play a critical role in immune responses and neural repair, influencing immune cell migration, activation, and function by regulating cellular interactions and signaling. Research has shown that after ischemic brain injury, the interaction between neurons and glial cells is regulated by adhesion molecules, especially ICAM-1 and VCAM-1 [55]. After CI, the inflammatory response in the brain can lead to upregulation of the expression of these adhesion molecules, thereby promoting the migration of immune cells to the site of injury [56]. For example, the absence of Poldip2 can significantly reduce the expression of adhesion molecules in the ischemic brain, thereby reducing the infiltration of immune cells, indicating that Poldip2 plays an important role in cerebral vascular inflammation and immune cell recruitment [56].

Adhesive molecules also participate in the process of neural repair. Research has found that the expression of adhesion molecules not only affects the migration of immune cells, but is also closely related to the regeneration of neurons. After CI, neurons promote nerve regeneration and functional recovery by upregulating adhesion molecules and interacting with surrounding cells. In a mouse model, inhibition of L1CAM results in a decrease in regeneration ability after nerve injury, highlighting the importance of L1CAM in nerve repair [57]. In peripheral nerve injury repair, the expression and functional changes of adhesion molecules directly affect the recruitment and activation of immune cells, thereby regulating inflammatory responses and promoting nerve regeneration [58]. For example, endothelial cells regulate the migration and localization of white blood cells by expressing adhesion molecules and chemokines, which is of great significance for restoring the function of damaged nerves [59]. These findings suggest that adhesion molecules have dual functions in regulating immune responses and promoting nerve repair, and may become targets for future treatments of nerve injuries.

At present, therapeutic strategies targeting adhesive molecules are constantly developing. Researchers are exploring novel therapies such as small-molecule inhibitors and monoclonal antibodies to specifically target adhesion molecules, thereby reducing nerve damage caused by CI [60]. For example, calcium channel blockers, such as pimozide and mibefradil, have been shown to effectively prevent CI, and their mechanism involves inhibition of calcium channels, thereby reducing neuronal apoptosis and inflammatory response [61]. Monoclonal antibodies targeting cell adhesion molecules, such as VCAM-1, have been shown to effectively reduce the infiltration of inflammatory cells, thereby reducing the degree of damage after CI [62]. In addition, adhesive molecules also provide new targets for nanomedicine delivery systems, improving therapeutic efficacy by enhancing targeting and drug biocompatibility [63]. For example, using nanoparticles to target inflammation-activated endothelial cells can effectively deliver drugs and improve therapeutic efficacy [64].

## 4 POST-ISCHEMIC CHEMICAL SIGNALING PATHWAYS: THE ROLE OF CYTOKINES AND CHEMOKINES

 After CI, cytokines and chemokines have an immeasurable role in tissue injury and repair. Cellular damage caused by CI is often accompanied by the activation of inflammatory reactions, which promote the release of various cytokines. These factors not only promote the infiltration of inflammatory cells into the damaged area, but also exacerbate cell apoptosis and necrosis by activating signaling pathways, such as the phosphatidylinositol 3 kinase/protein kinase B (PI3K/Akt) and nuclear factor kappa-light-chain-enhancer of activated B cells (NF-κB), further expanding tissue damage [65, 66]. At the same time, chemokines are rapidly released, guiding immune cells to migrate to CI areas by binding to their receptors, thereby enhancing local inflammatory responses and participating in the clearance and repair processes of damaged tissues [67, 68]. The interaction of cytokines and chemokines also influences the process of regeneration and repair. Research studies have shown that certain cytokines can promote the migration and differentiation of stem cells, thereby accelerating tissue repair [69].

Therefore, understanding the roles of cytokines and chemokines in post-ischemic chemical signaling pathways not only helps to unravel the mechanisms of ischemic injury, but also provides potential targets for the development of new therapeutic approaches.

### 4.1 Changes in Cytokines and Chemokines after Ischemia: TNF-α, IL-1β, and IL-6

 Tumor Necrosis Factor Alpha (TNF-α), interleukin-1 beta (IL-1β), and interleukin-6 (IL-6) are major pro-inflammatory cytokines that are rapidly released in the early stages after ischemic injury, promoting inflammatory responses and exacerbating tissue damage [70]. Research has shown that TNF-α promotes an inflammatory response after ischemia by activating various signaling pathways (such as the NF-κB pathway), leading to cell apoptosis and tissue necrosis [71]. Currently, TNF-α inhibitors are available for targeted therapy [72]. IL-1β, as a potent pro-inflammatory cytokine, not only promotes the infiltration of inflammatory cells after ischemia, but also exacerbates local inflammatory responses by inducing the release of other cytokines [73]. IL-1 receptor antagonists have been shown to reduce infarct volume in animal models [74].

The role of IL-6 is equally significant, and it is considered a key regulator of acute and chronic inflammation. The release of IL-6 mainly comes from various cells in the brain, including astrocytes, microglia, and neurons [75]. These cells activate the expression of IL-6 through different signaling pathways after CI, such as janus kinase/signal transducers and activators of transcription (JAK/STAT) signaling pathway, NF-κB signaling pathway, and mitogen-activated protein kinase (MAPK) signaling pathway, thereby affecting the function of surrounding cells and the stability of neural networks [76, 77]. IL-6, through its receptor-mediated signaling pathway, not only promotes inflammatory responses, but may also promote neuronal survival and regeneration by activating relevant protective signaling pathways [78]. In the mouse model of CI, the level of IL-6 is significantly increased, and its concentration is positively correlated with the severity of the disease [79]. IL-6 blockers have been studied to reduce inflammation-related CI injury [80].

After CI, changes in cytokines not only affect the local inflammatory response, but may also influence the systemic immune status. It has been found that elevated TNF-α, IL-1β, and IL-6 are associated with the activation and migration of immune cells following ischemic injury, and collectively, these factors are involved in the immune response after CI, affecting the organism's repair process [81]. The relationship between the quantity and time of TNF-α, IL-1β, and IL-6 release trends at different time points is shown in Fig. (**2**). Therefore, modulating the release and action of these pro-inflammatory cytokines may become a new approach in the treatment of ischemic injury, which can effectively reduce the inflammatory response and promote tissue repair [82].

### 4.2 Signaling Molecules Regulate Neuroinflammation, Apoptosis, and the Later Repair Process in CI

 In the pathological process of the nervous system, neuroinflammation, apoptosis, and the subsequent repair process are closely interconnected, and signaling molecules play a crucial role in this series of events. Neuroinflammation is caused by a variety of factors, such as infection, trauma or neurodegenerative diseases, and microglia and astrocytes are the main effector cells of neuroinflammation, which regulate the immune response of the nervous system by releasing cytokines, chemokines, and other signaling molecules [83].

Apoptosis is a major mechanism by which the nervous system responds to damage, and excessive apoptosis leads to neuronal loss, thereby exacerbating neurological dysfunction. Research studies have shown that the activated Janus kinase 2/signal transducer and activator of transcription 3 (JAK2/STAT3) signaling pathway act in conjunction with other transcription factors, such as NF-κB and mammalian target of rapamycin (mTOR), to regulate the process of apoptosis, further exacerbating the effects of neuroinflammation [83, 84]. In addition, miRNAs also play a significant regulatory role in neuroinflammation and apoptosis, affecting neuronal survival and death by modulating components of relevant signaling pathways [83].

Signaling molecules also have an irreplaceable role in the later repair process. Signal molecules during the repair process, such as silent mating type information regulation 2 homolog-1 (SIRT1), inhibit NF-κB activity through deacetylation, thereby reducing neuroinflammation and promoting neuronal survival [85]. In addition, itaconate, an immunomodulatory metabolite, inhibits neuroinflammation and modulates the immune response in the central nervous system, demonstrating potential in the treatment of neuroinflammation-related diseases [86].

### 4.3 The Dual Role of Cytokines in Microvascular Remodeling and Neuroprotection

Cytokines play a regulatory role in microvascular remodeling and neuroprotection. Microvascular remodeling refers to changes in the structure and function of microvessels, a process often closely associated with inflammatory responses. Studies have shown that inflammatory cytokines play a promoting role in microvascular remodeling, affecting microvascular permeability and hemodynamics by regulating the function of endothelial cells [87]. For example, TNF-α can induce an inflammatory response in endothelial cells, leading to changes in microvascular structure and dysfunction, which in turn, affect neuronal blood supply and nutrition [88].

On the other hand, certain cytokines also have neuroprotective effects. For example, interleukin-10 (IL-10) is thought to play a role in inhibiting inflammatory responses and promoting microvascular repair [89]. The release of IL-10 can alleviate nerve damage, promote neuronal survival, and functional recovery by inhibiting the production of pro-inflammatory cytokines [90]. In addition, regulatory T cells (Tregs) have been found to enhance neuroprotection and alleviate ischemia-reperfusion injury through the secretion of cytokines, such as IL-10 and transforming growth factor β (TGF-β) [91]. The relationship between the number and time of IL-10 release trends at different time points is shown in Fig. (**2**).

Cytokine interactions appear to be particularly complex during microvascular remodeling. The regeneration and remodeling of microvessels after ischemia are closely related to the dynamics of cytokines [92]. It has been shown that after ischemia-reperfusion, the release of endogenous cytokines promotes microvascular regeneration and improves local blood flow, thereby having a protective effect on neurons [93]. However, excessive cytokine release may lead to sustained inflammatory response, thereby exacerbating nerve damage. Table **2** lists the major cytokines associated with CI, including related functions, mechanisms, and research progress.

## 5 ROLE OF EXOSOMES AND MICROVESICLES IN NEUROPROTECTION AND REGENERATION

Exosomes and microvesicles, as mediators of intercellular communication, have attracted widespread attention in the field of neuroprotection and regeneration. Research has shown that extracellular vesicles from mesenchymal stem cells (MSCs) can carry various active molecules, regulate neuroinflammation, promote neuronal survival and functional recovery, and effectively alleviate CI and brain injury [94, 95]. Meanwhile, extracellular vesicles derived from microglia can also be taken up by neurons and inhibit apoptosis by regulating signaling pathways [96, 97]. Microcapsules enhance the neuronal microenvironment, promoting repair and regeneration by transporting growth factors and regulating inflammatory responses [98, 99]. These findings offer new insights into the treatment of neurological diseases.

In conclusion, the mechanism of action of exosomes and microvesicles in neuroprotection and regeneration is complex and involves intercellular signaling, immune regulation, and the regulation of cell growth and survival. The formation process of exosomes (30-150 nm) and microvesicles (100-1000 nm) and their mechanism of action after crossing the BBB are shown in Fig. (**3**). Exosomes are generated in multivesicular bodies through endocytosis and released outside the cell, whereas microvesicles are formed directly from the cell membrane. These extracellular vesicles can carry miRNAs (*e.g*., miR-21-5p) across the BBB, and after entering the central nervous system, they activate multiple signaling pathways, inhibit neuronal apoptosis, and promote the release of anti-inflammatory factors and vascular remodeling, thereby maintaining neuronal health. Future studies will further elucidate the specific mechanisms of action of these extracellular vesicles and explore their potential application in the treatment of clinical neurological diseases.

### 5.1 Biological Functions of Exosomes and Microvesicles: Signal Carriers between Cells

Exosomes and microvesicles have garnered significant attention in recent years as carriers of intercellular signaling. Exosomes are small lipid bilayer vesicles formed by cells through endocytosis, typically ranging in diameter from 30 to 150 nanometers [100]. Microvesicles are formed by the protrusion of the cell membrane, usually larger than exosomes, with a diameter range of 100-1000 nanometers [101]. They participate in intercellular signaling and play a role in both cellular physiological and pathological processes [102, 103]. The release of exosomes and microvesicles is regulated by various factors, including cell type, environmental conditions, and cellular state, resulting in significant differences in their functions under different physiological and pathological states [104, 105].

Notably, exosomes and microvesicles are not only carriers of intercellular information transmission, but also considered as potential biomarkers and therapeutic carriers. They are capable of carrying tumor-specific markers, providing the possibility of early diagnosis, and due to their good biocompatibility and low immunogenicity, exosomes and microvesicles have shown promising applications in drug delivery systems [106, 107].

### 5.2 Role in CI: Molecular Communication by Carrying RNA, Proteins, and Lipids

Intercellular molecular communication is essential during the pathological process of CI. In particular, extracellular vehicles (EVs) and other types of cellular secretions have significant functions in neuroprotection and repair [108]. These vesicles are capable of carrying a wide range of biomolecules, including RNA, proteins, and lipids, as well as anti-inflammatory and pro-growth factors, which are involved in regulating the inflammatory response after CI and promoting cell survival and functional recovery [79]. For example, exosomes from adipose-derived stem cells (ADSC-exosomes) are able to attenuate the neuroinflammatory response after CI by translocating miR-21-5p, a process that involves the regulation of the phosphatidylinositol-3-kinase regulatory subunit 1/phosphatidylinositol 3-kinase/protein kinase B (PIK3R1/PI3K/AKT) signaling pathway [109]. In addition, exosomes from neural stem cells can inhibit the caspase 2 (CASP2) signaling pathway *via* miR-150-3p to enhance neuroprotection after CI, thereby inhibiting neuronal apoptosis and promoting neuronal proliferation after brain injury [110]. Exosomes from astrocytes can attenuate neuronal death and apoptosis *via* miR-92b-3p, which can also attenuate neuronal death and apoptosis [110]. Moreover, astrocyte exosomes are able to inhibit neuronal apoptosis by regulating autophagy and miR-7670-3p/SIRT1 *via* the circular RNA circSHOC2 [110]. The lipid components of extracellular vesicles have been suggested to play a role in regulating cell signaling and maintaining cell membrane integrity [110].

Studies have also shown that the role of non-coding RNAs (*e.g*., circular RNAs and long non-coding RNAs) in CI cannot be ignored. These RNAs may play a critical role in the repair process of ischemic injury by regulating gene expression and cell fate. For example, circular RNAs are abundantly expressed in brain tissue and may be closely associated with neurodevelopment and disease progression [111]. The changes in the expression of long non-coding RNAs after CI have been suggested to be closely related to the recovery of brain function and may represent a future therapeutic target [112].

### 5.3 The Potential of Exosomes in Modulating Immune Responses and Promoting Neuroprotection and Nerve Regeneration

Exosomes, as carriers of intercellular communication, now show great potential in regulating immune responses and promoting neuroprotection and nerve regeneration. In the immune response, exosomes are able to modulate the strength and nature of the immune response by influencing the function of immune cells. For example, exosomes secreted by immune cells can mediate the interaction between adaptive and innate immunity and regulate tumor progression and metastasis [113]. Exosomes can play a crucial role in the immunotherapy of various diseases by regulating cytokine production and signaling pathways, thereby influencing the homeostasis of the immune system [114].

The role of exosomes in neuroprotection and nerve regeneration should not be overlooked as well. Exosomes from MSCs have been shown to promote neuronal survival, regeneration, and neuroplasticity by transporting specific miRNAs and growth factors [115]. Studies have shown that MSCs-derived exosomes significantly improve neurological function, reduce cell death, and enhance neuroprotection by modulating relevant signaling pathways, such as the pTEN-induced putative kinase 1/parkin (PINK1/Parkin) pathway, in models of ischemic brain injury [116, 117]. Exosomes have shown potential to promote nerve regeneration after spinal cord injury and can improve functional recovery by modulating the inflammatory response and promoting the differentiation of neural stem cells [118, 119].

With the in-depth study of the repair mechanism after CI, exocytosis has gradually attracted the attention of the scientific community. Exosomes have low immunogenicity and good biocompatibility, and can effectively penetrate the BBB [120]. Following CI, exocytosis plays a crucial role in protecting neuronal cells and enhancing the cerebral microenvironment by regulating inflammatory responses, promoting nerve regeneration, and facilitating vascular remodeling, among other mechanisms [120]. For example, M2 microglia-derived exosomes are able to reduce neuronal apoptosis and improve functional recovery after ischemic brain injury by releasing miR-124 [121]. Some major intercellular communications are listed in Table **3**, including the source of exosomes, components, function, mechanism, and experimental type.

However, exosomes also face several challenges in clinical applications. Firstly, the source and preparation method of exosomes can affect their biological activity and therapeutic effects. Studies have shown that exosomes from different sources have differences in composition and function. For example, there are significant differences in the ability of exosomes from healthy individuals and patients with myocardial infarction to repair damaged myocardial cells [122]. Secondly, the *in vivo* distribution and clearance rate of exosomes are also major factors affecting their efficacy. Although exosomes exhibit good targeting properties, their concentration and duration of action still require further optimization in the post-ischemic microenvironment [123]. Additionally, the low yield and production cost of exosomes are also factors limiting their clinical application [124].

## 6 GAP JUNCTIONS AND NANOTUBES IN ISCHEMIC STRESS: HIGHWAYS OF METABOLISM AND SIGNAL EXCHANGE

Intercellular communication is crucial during ischemic stress. Connection structures, such as gap junctions and nanotubes, provide "high-speed channels" for the rapid transmission of signals and metabolites between cells. Gap junctions form channels by connecting adjacent cell membranes, allowing for the direct exchange of small molecules, ions, and signaling molecules, thereby regulating cellular physiological functions [125, 126]. As a novel communication method, nanotubes can transmit organelles and signaling molecules over longer distances, promoting collaborative responses between cells [127]. These structures play a crucial role in regulating cellular responses to ischemic injury.

Therefore, investigating the role of gap junctions and nanotubes in ischemic stress not only helps to understand the dynamics of intercellular signaling and metabolism but also provides new insights for the development of therapies for ischemic injury.

### 6.1 Gap Junctions and the Structure and Function of Nanotubes: Direct Communication Pathways between Cells

Gap junctions are channels composed of connexins that allow small molecules and ions to be directly transferred between adjacent cells, thereby achieving rapid intercellular signaling [128]. This structure is not only significant in normal physiological processes, such as cell growth, differentiation, and metabolic regulation, but also in pathological conditions. However, it is also associated with many pathological states, including the occurrence and development of tumors [129]. For example, in urinary tract cancer, dysfunction of gap junctions is believed to be associated with tumor progression and metastasis [129].

Nanotubes are another means of intercellular communication, particularly in the context of cellular exosomes. Research has shown that cells can transmit intracellular signaling molecules, proteins, RNA, and other substances to neighboring cells through nanotubes, thereby affecting their function and behavior [130]. This intercellular material exchange is not limited to small molecules; nanotubes can also deliver larger molecules, enhancing intercellular signaling and information sharing [130].

### 6.2 Role in Ischemic Stress: Promoting the Rapid Spread of Ions, Metabolites, and Signaling Molecules

In the presence of ischemic stress, the metabolic demands of cells increase, and the function of gap junctions and nanotubes becomes particularly critical. Under ischemia, cells rapidly exchange protective and harmful metabolites through gap junctions to maintain cell survival and function [125]. It has been demonstrated that changes in cell membrane potential and the activation of ion channels are key factors in facilitating intracellular signaling following ischemia [131]. For example, activation of voltage-gated calcium channels leads to a rapid increase in intracellular calcium ion concentration, which not only affects cellular metabolic activities, but also triggers the activation of a series of signaling pathways, including oxidative stress and inflammatory responses [132].

Equally important is the rapid clearance of metabolites. Under ischemic conditions, alterations in cellular metabolism can lead to inadequate energy supply, which in turn affects cell survival and function. It has been found that intracellular levels of reduced glutathione (GSH) significantly decrease after ischemia, which is closely related to the level of oxidative stress in cells [133]. After ischemia, cells regulate the response of surrounding cells by releasing EVs, which contain a variety of biologically active substances that transmit signals between cells and promote adaptive responses [134].

In terms of signal molecule propagation, cells in ischemia enhance the rapid transmission of signaling molecules by altering the fluidity and permeability of the cell membrane. This mechanism is not limited to the propagation of ions and metabolites, but also involves the release of cytokines and chemokines, molecules that rapidly attract immune cells to the damaged area after ischemia, thereby promoting inflammatory responses and tissue repair [135]. For example, inflammatory factors, such as TNF-α and IL-1β, released after ischemia, are able to activate the stress response of peripheral cells, thereby accelerating the repair process [136].

### 6.3 Association of Gap Junctions and Nanotubes with Metabolic changes after CI

The changes in intercellular function of gap junctions after CI may have a profound impact on the recovery from brain injury. Studies have shown that gap junction proteins, such as connexins and innexins, exhibit significant changes after CI that are closely associated with cellular metabolic dysregulation and inflammatory responses. For example, connexin 43 (Cx43) has been found to regulate intercellular electrical signaling after ischemia, thereby affecting neuronal survival and function [137]. In addition, the remodeling of gap junctions caused by ischemia is associated with neuronal death and dysfunction, which suggests that restoring normal gap junction function may be a strategy for treating the consequences of CI [138].

Nanotubes are widely used in the biomedical field due to their excellent physical and chemical properties. Research studies have shown that nanotubes can improve cellular metabolism and survival after CI by enhancing the formation and function of gap junctions. For example, by adjusting the surface properties of nanotubes, intercellular substance exchange can be facilitated, thereby improving cellular adaptation to the ischemic environment [139]. In addition, the potential of nanotubes in neuroprotection and the promotion of BBB restoration has also been demonstrated, providing new ideas for treatment after CI [140].

Clinical studies have gradually recognized the function of gap junctions and nanotubes in metabolic changes after CI. By studying models of ischemic brain injury, it was found that drugs modulating gap junctions could significantly improve neurological function and reduce cell death [141]. Meanwhile, the application of nanotubes as drug delivery carriers can improve drug bioavailability in brain tissue and enhance therapeutic effects [142]. These studies indicate that the regulation of gap junctions and nanotubes is not only important in basic research but also holds promising prospects for clinical applications, providing new insights for treating metabolic changes after CI.

## 7 APPLICATION OF ADVANCED TECHNOLOGY IN DECODING INTRACELLULAR COMMUNICATION AFTER ISCHEMIA

In recent years, with the rapid development of biomedical technology, the application of advanced techniques in decoding intracellular communication after ischemia has gradually become a hot research topic. Altered intracellular communication following ischemic injury is a contributing factor to cellular dysfunction and death; therefore, a deeper understanding of these communication mechanisms is essential for the development of new therapeutic strategies. Modern technologies, such as scRNA-seq, imaging techniques, and nanotechnology, provide unprecedented tools for studying intercellular signaling and interactions.

The advent of scRNA-seq has enabled researchers to analyze gene expression changes in cells following ischemia at the single-cell level. This technology not only reveals cell-type-specific signaling pathways but also identifies key genes and regulatory networks that are active under ischemic conditions, thereby providing new insights into the complexity of intracellular communication [143]. In addition, mass spectrometry imaging enables real-time monitoring of changes in intracellular metabolites, which is particularly critical for studying cellular metabolic reprogramming after ischemia and its impact on cellular communication. By quantifying intracellular signaling molecules, researchers can gain a deeper understanding of how cells regulate their communication through metabolic changes following ischemia [144]. The application of nanotechnology has revolutionized the study of intracellular communication by enabling targeted delivery of drugs and genes to modulate intercellular signaling. For example, the use of engineered exosomes as nanocarriers can effectively deliver therapeutic molecules and facilitate intercellular communication and interactions [145].

**Fig. (4):**
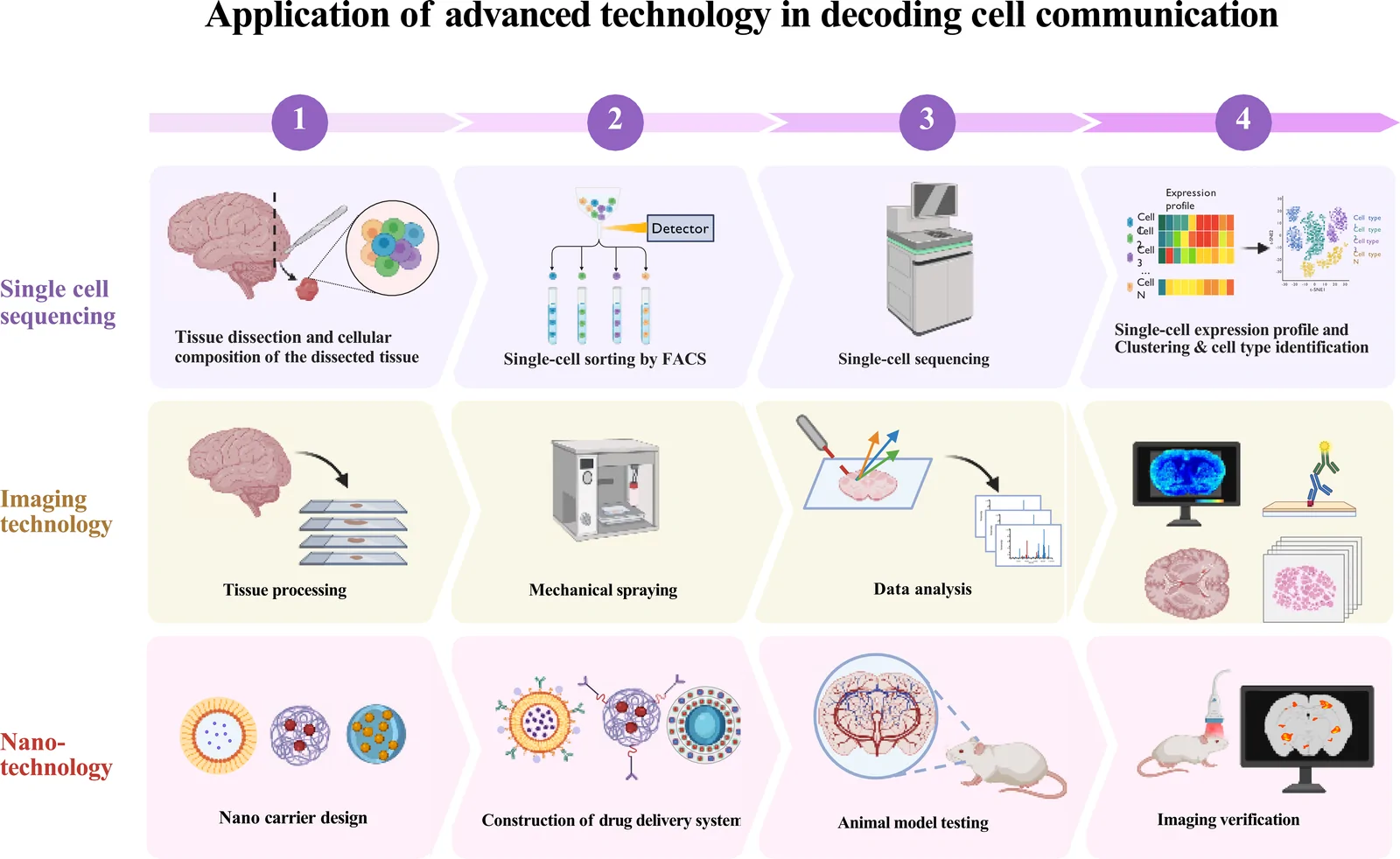
Applications of advanced technologies in decoding intercellular communication.

The application of advanced technologies to decipher intracellular communication after ischemia not only enhances our understanding of pathophysiological mechanisms, but also provides new targets for clinical treatment (Fig. **4**). As technology continues to advance, it is expected that more precise and effective therapeutic options will be available in the future to improve the quality of life of patients with ischemic diseases.

### 7.1 Application of Existing Techniques in the Study of Intracellular Communication

In recent years, scRNA-seq has demonstrated great potential in deciphering intracellular communication. scRNA-seq can provide transcriptomic information about individual cells, enabling researchers to gain insight into cellular heterogeneity and gene expression dynamics. In ischemic research, single-cell sequencing technology is widely used to analyze cellular responses and adaptation mechanisms in ischemic diseases. For example, researchers used scRNA-seq to investigate the transcriptome changes in microglia after ischemic stroke and found that these cells underwent significant phenotypic changes following ischemia, with specific gene expression patterns associated with neuroprotective effects [146]. In addition, single-cell sequencing has been used to analyze the regenerative ability of myocardial cells after myocardial ischemia, revealing functional differences among different cell populations during ischemic recovery [147]. These cases illustrate the role of scRNA-seq in elucidating intracellular communication and the biological responses that follow ischemia. The amount of data generated by scRNA-seq is vast and complex, and modern bioinformatics tools and algorithms are widely used to process and analyze scRNA-seq data. These tools can visualize high-dimensional data in a low-dimensional space, helping researchers to intuitively observe the distribution and heterogeneity of cellular populations. These advances in data parsing not only improve the efficiency of research, but also provide a reference basis for clinical applications.

Imaging technology has also played a role in intracellular communication research. Modern imaging technologies, such as ultrasound imaging, Magnetic Resonance Imaging (MRI), and Mass Spectrometry Imaging (MSI), provide real-time visualization of cells and tissues. Advances in ultrasound imaging techniques, such as contrast-enhanced ultrasound (CEUS) and elastography, enable the *in vivo* observation of cellular interactions and signaling. With these imaging techniques, researchers can monitor the dynamics of cells in real-time and analyze how cells signal through structures, such as extracellular vesicles, thereby revealing the mechanisms of intercellular communication [148, 149]. The metabolic state of cells changes significantly after ischemia, and MSI can be used to analyze the spatial distribution of these metabolites. For example, it has been shown that MSI can reveal changes in key metabolites, such as adenosine triphosphate (ATP) and lactate in cells after ischemia, and the distribution of these metabolites is closely related to the energy status and viability of cells [150, 151]. By imaging and analyzing these metabolites, researchers are able to better understand the effects of ischemia on cellular function and how cells respond to ischemic injury through metabolic reprogramming [152, 153].

Nanotechnology also shows unique promise for applications in cellular communication research. Nanomaterials can be designed to target the interior of cells and influence cellular behavior by modulating intracellular signaling pathways. For example, nanoparticles can be used as drug delivery systems to precisely deliver therapeutic drugs to specific cell types, thereby modulating intercellular signaling and communication. Nanotechnology can be used to develop novel biosensors to monitor signaling molecules inside and outside the cell, which provides new perspectives for understanding cellular interactions [154, 155].

### 7.2 Deeply Understanding the Intercellular Communication Network in CI through Multiple Technologies

CI is a complex pathological state that involves interactions and communication between multiple cell types. In recent years, with the development of scRNA-seq, imaging technology, and other advanced technologies, researchers have been able to explore the dynamics of intercellular communication networks during CI in greater depth. Through these technologies, scientists can analyze intercellular signaling mechanisms at the single-cell level, revealing intercellular communication networks and identifying the key cell types involved in CI, as well as the molecular basis of their interactions. Experiments have demonstrated significant alterations in miRNAs and surface proteins in the exosomes of microvascular endothelial cells following ischemia, which are associated with cell proliferation and angiogenesis [156]. These exosomes not only play a role in the pathological process of CI but may also serve as new therapeutic targets. In addition, studies have shown that intercellular signaling pathways are indispensable in the recovery process after CI, and aberrant activation of these pathways may lead to cell death or dysfunction [157].

Although the accuracy and efficiency of intercellular communication may be affected by a variety of factors after ischemia, including cell type, microenvironment, and pathological state [158], by integrating different single-cell technologies and computational biology approaches, researchers were able to construct a panorama of intercellular communication in CI, identifying potential therapeutic targets and biomarkers [159]. The application of advanced technologies not only provides powerful tools for a deeper understanding of the intercellular communication network in CI, but also may advance the development of early diagnosis and personalized treatment of CI.

### 7.3 Limitations of the Technology and Future Directions

Despite significant progress in the application of advanced technologies, several limitations persist in the study of decoding post-ischemic intracellular communication. First, the complexity and high cost of data analysis of existing technologies limit their widespread use in clinical practice. For example, although scRNA-seq can provide high-resolution data at the cellular level, its data processing and analysis are complex and require a high level of bioinformatics support [160].

Second, the limitations of existing technologies are primarily due to a lack of dynamic and temporal resolution. Although the application of advanced technologies, such as scRNA-seq and spatial transcriptomics, in recent years has provided new perspectives on understanding cellular communication, there are still challenges in capturing the dynamic communication between cells using these technologies. For example, although scRNA-seq can provide transcriptomic information of cells at specific time points, its inability to monitor the dynamic signaling process between cells in real-time leads to an inadequate understanding of the timeliness of cellular communication [161]. In addition, existing techniques often rely on static samples, which makes it difficult for researchers to access the dynamic changes of cells in different physiological or pathological states.

The lack of temporal resolution also limits a comprehensive understanding of cellular communication networks. Rapid transmission of signals and feedback mechanisms are indispensable in cellular communication, yet conventional imaging techniques often fail to capture these transient signals with sufficient temporal resolution. For example, although high-resolution calcium imaging techniques can monitor the dynamics of intracellular calcium ions, when dealing with non-attached cells, the mechanical displacement of the cells may affect the spatial resolution of the imaging, thus compromising the accurate analysis of intercellular communication [162]. Therefore, although technological advances have provided new tools for cellular communication studies, the lack of dynamic and temporal resolution remains a pressing issue.

To overcome these limitations, researchers are exploring new techniques and approaches. For example, real-time monitoring using high-throughput biosensor platforms can analyze cell-secreted proteins at the single-cell level, thus revealing dynamic intercellular communication processes [163]. Additionally, the development of more efficient computational tools, such as scSeqComm, can aid researchers in identifying and quantifying intercellular signaling from scRNA-seq data [164]. The application of these emerging technologies is expected to improve the dynamics and temporal resolution of cellular communication studies, provide more precise tools and methods for understanding the complex signaling networks between cells. Table **4** lists the application comparison of technologies in the CI immunization network, which can be comprehensively resolved through the combination of multi-omics technologies, such as transcriptomics, proteomics, and metabolomics [165]. On the other hand, the application of artificial intelligence and machine learning will help to improve the efficiency and accuracy of data analysis, leading to a better understanding of the dynamic interactions between cells [166].

## 8 INNOVATIVE THERAPEUTIC STRATEGIES TARGETING INTERCELLULAR COMMUNICATION PATHWAYS

Innovative therapeutic strategies targeting intercellular communication pathways have gradually become a popular research direction in modern medical research, especially in the treatment of cancer and other complex diseases. Intercellular communication is a fundamental process in cell biology that involves cells exchanging information through direct contact or secreting signaling molecules [167]. For example, exosomes derived from adipose-derived stem cells (ASC-derived extracellular vesicles, ASC-EVs) can alleviate neuronal damage after ischemia-reperfusion by transferring miR-26a, demonstrating their potential as therapeutic agents [168].

Furthermore, the interaction between endothelial cells (ECs) and pericytes (PCs) is irreplaceable in angiogenesis and stabilization. Studies have shown that signaling pathways between ECs and PCs, such as the VEGF and platelet-derived growth factor (PDGF) pathways, are key links in intercellular communication [169]. Interference with these signaling pathways may lead to abnormal blood vessel formation, which in turn can lead to a variety of diseases, including cardiovascular disease and cancer [170, 171]. Therefore, therapeutic options targeting these signaling pathways are being developed to improve the prognosis of vascular-related diseases [172].

In summary, innovative therapeutic strategies targeting intercellular communication pathways provide new ideas and approaches for the treatment of a wide range of diseases. By examining the signaling mechanisms between cells, scientists can identify new drug targets and develop more effective therapeutic regimens to enhance the clinical prognosis of patients. As research continues, it is expected that further breakthroughs in precision medicine and individualized treatment will be made in the future, advancing the progress of disease treatment.

### 8.1 Therapeutic Targets based on Intercellular Communication

Intercellular communication plays a major role in the growth, development, and immune response of organisms. In recent years, intercellular communication mechanisms, including adhesion molecules, chemical signaling pathways, exosomes, and gap junctions, have been found to have a significant impact on the onset and progression of disease, and have become potential therapeutic targets [173]. Adhesive molecules are proteins expressed on the surface of cells, mainly responsible for intercellular adhesion and signal transmission. In the context of CI, extracellular vesicles affect the inflammatory response and neuroprotective mechanisms after CI by regulating signal transmission between neurons, glial cells, and endothelial cells [174, 175].

The chemical signaling pathway mediates intercellular signaling through molecules, such as cytokines, chemokines, and growth factors. After CI, the survival of neurons is closely related to the function of adhesion molecules. Endothelial cell damage leads to the breakdown of the BBB, promoting the infiltration of inflammatory cells and neuronal damage [174]. During this process, the upregulation of adhesion molecules, such as ICAM-1 and VCAM-1, promotes the adhesion and migration of inflammatory cells, thereby exacerbating neuronal damage [176].

Exosomes are nanoscale membrane vesicles secreted by cells that can carry a variety of bioactive molecules and participate in intercellular messaging. The pathophysiological mechanism of CI is complex, involving the regulation of multiple chemical signaling pathways [177]. Research has shown that after CI, both intracellular and extracellular signaling pathways are significantly affected, primarily including the PI3K/ Akt, MAPK, NF-κB, and Wnt/β-Catenin pathways [178]. These pathways play important roles in cell survival, apoptosis, inflammatory response, and neural regeneration [179].

Gap junctions are important structures for direct cell-to-cell communication and are mainly composed of connexins. These connexins form complete gap junction channels by forming connexons [180]. These channels enable the free exchange of ions and small molecules between neighboring cells, thereby playing a crucial role in maintaining the coordination of cellular functions and the overall homeostasis of tissues [181]. Between vascular endothelial cells and peripheral smooth muscle cells, gap junctions not only ensure direct cell-to-cell communication but also play a crucial role in the behavioral synchronization of the vascular tree [182]. Some major intercellular communications are listed in Table **5**, including communication pathways, molecular basis, function, and effects of CI.

### 8.2 The Challenge of Clinical Translation: A Bridge from Basic Research to Therapeutic Applications

Clinical translation is a central aspect in the field of medical research, aiming to translate discoveries from basic research into effective clinical treatment protocols. However, this process faces several challenges. Firstly, the divide between basic research and clinical application is often reflected in the reproducibility and clinical applicability of research results. For example, many treatments that have shown promising results in animal models have failed to achieve the expected results in human clinical trials [183]. Additionally, there are challenges in the design and implementation of clinical trials, particularly in selecting suitable patient populations and developing effective efficacy assessment metrics [184]. These problems not only affect the speed of developing new therapies but also limit their application in actual clinical settings.

Secondly, ethical issues are also a critical challenge to clinical translation. In the process of accelerating the translation of research results, ensuring the scientific and ethical nature of the research and avoiding the relaxation of scientific methods and regulatory requirements caused by the rush to achieve results is an urgent issue [185]. For example, in the process of drug development, researchers must find a balance between ensuring patient safety and access to effective treatments, which places greater demands on the design of clinical studies.

Furthermore, limitations in technology and resources have also hindered clinical translation. Many emerging therapies, particularly gene therapy and cell therapy, have shown promising results in the laboratory but face complex manufacturing and quality control challenges in clinical applications [186]. The lack of validated biomarkers makes it difficult for clinicians to select appropriate treatment options [187].

Finally, interdisciplinary collaboration and communication have a decisive position in overcoming challenges in clinical translation. Effective communication between the researchers and clinicians can help researchers better understand clinical needs, enabling them to consider the feasibility of clinical application at the study design stage [188]. Meanwhile, the use of modern technologies, such as big data and artificial intelligence, can enhance the efficiency and accuracy of research, providing new ideas and methods for clinical translation [189].

### 8.3 Future Research Directions: Developing More Targeted and Effective Cell-cell Communication Targeted Therapy Methods

Intercellular communication is crucial in maintaining normal physiological functions and responding to pathological states in organisms. In recent years, with the advancement of single-cell RNA sequencing (scRNA-seq), researchers have gained a deeper understanding of the mechanisms underlying intercellular communication. For example, scRNA-seq revealed that specific cell types, such as macrophages and microglia, regulate local inflammatory responses and repair processes by secreting cytokines and interacting with extracellular vesicles after ischemia [190]. The development of computational tools, such as CellChat, has enabled researchers to quantitatively infer and analyze intercellular communication networks from scRNA-seq data, providing strong support for the design of targeted therapies [191].

Future research directions should focus on the development of more targeted intercellular communication-targeted therapeutics. A detailed analysis of intercellular communication networks can identify key ligand-receptor interactions, providing precise targets for therapies in various disease states. For example, in cancer research, communication between tumor cells and microenvironmental cells is recognized as a factor that influences tumor progression and treatment resistance [192]. By constructing a comprehensive database of ligand-receptor interactions, researchers can more effectively decipher intercellular communication in the tumor microenvironment, thus providing new targets for anti-cancer drug development.

Abnormal intercellular communication has significant implications in various diseases, such as Parkinson's disease and renal fibrosis [193, 194]. A detailed study of intercellular signaling pathways in these diseases can help identify potential therapeutic targets, thereby facilitating the development of personalized therapies. With the continuous progress of bioinformatics and systems biology, future studies will increasingly focus on elucidating the complex roles of intercellular communication in disease development through integrated multi-omics analyses, thereby providing new insights for targeted therapies.

### CONCLUDING REMARKS AND FUTURE PERSPECTIVES

CI, as a complex neurological disease, has a core pathological mechanism involving multiple cell types and their communication networks. Recent studies have demonstrated that the expression of intercellular communication during injury onset and repair in CI involves a range of pathways, including cytokines, exosomes, adhesion molecules, and gap junctions. An in-depth understanding of these mechanisms not only helps to reveal the molecular basis of CI, but also provides clues for the development of innovative therapeutic plans.

 When summarizing the current research progress, it becomes clear that the mechanisms of intercellular communication primarily involve interactions between neurons and glial cells, the release of cytokines, and the transmission of extracellular vesicles. The study of these mechanisms has revealed potential pathways for neuroprotection and repair after CI. However, existing research has mostly focused on a specific cell type or communication mode, lacking a systematic and comprehensive analysis. Future research should aim to integrate the communication mechanisms of different cell types, explore their synergistic effects in CI, and thus form a more complete pathological model.

In addition, with the advancement of technology, future research should focus on the following key points. The application of emerging technologies, such as single-cell sequencing and high-throughput screening, will provide more accurate tools for studying intercellular communication in CI. The introduction of these technologies not only helps us identify new biomarkers, but may also reveal potential intervention targets, thereby promoting the development of personalized therapy. Furthermore, the dynamic changes in intercellular communication during CI should be further revealed, especially the specific mechanisms at different pathological stages and time windows. By combining advanced technologies, such as single-cell sequencing and spatial genomics, it is possible to comprehensively analyze the cellular communication network and its spatiotemporal characteristics, providing precise information for targeted interventions.

Meanwhile, it remains a challenge in current research to strike a balance between different types of knowledge, despite the fact that various technologies and methods offer their own insights. Interdisciplinary collaboration is particularly important, and the cross-fertilization of biology, medicine, engineering, and data science will help drive the translation of basic research into clinical applications. In this process, establishing a standardized methodology and data sharing platform will facilitate the integration of findings from different studies and improve the reproducibility and reliability of research. Interdisciplinary collaboration will help decipher the complex pathological network of CI and develop more targeted intervention guidelines.

In terms of therapeutic applications, targeted therapies based on intercellular communication hold broad prospects, and future research directions should focus on developing potential therapeutic strategies, particularly exosome-based drug delivery systems. For example, the excellent biocompatibility and delivery ability of exosomes as carriers of intercellular information transfer make them potential drug delivery tools [195, 196]. By utilizing exosomes to transport therapeutic miRNAs or drug molecules, precision therapy can be achieved in specific regions of CI. Molecular drugs targeting cytokine and adhesion molecule pathways offer new possibilities for modulating inflammatory responses and promoting neural repair.

In conclusion, studies of intercellular communication have provided new perspectives on the pathological mechanisms and treatment of CI. However, many technical, resource, and ethical challenges still need to be overcome to translate these findings into clinical practice. With the continuous advancement of technology and the deepening of interdisciplinary collaboration, it is expected that in the future, more precise and efficient therapeutic guidance will be developed, bringing substantial benefits to patients with CI and further improving their quality of life.

## Figures and Tables

**Fig. (2):**
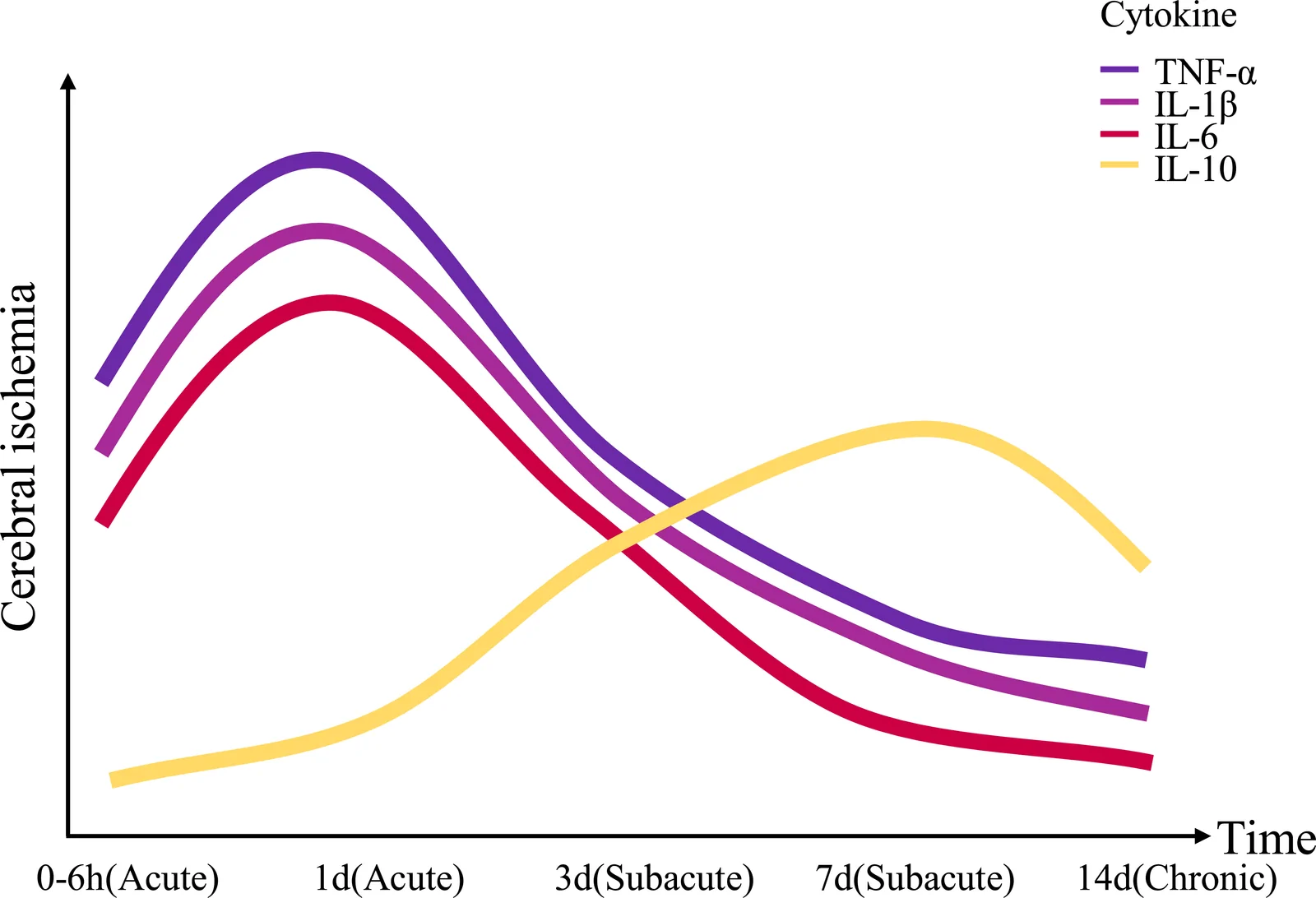
Temporal changes and roles of cytokines after CI.

**Fig. (3):**
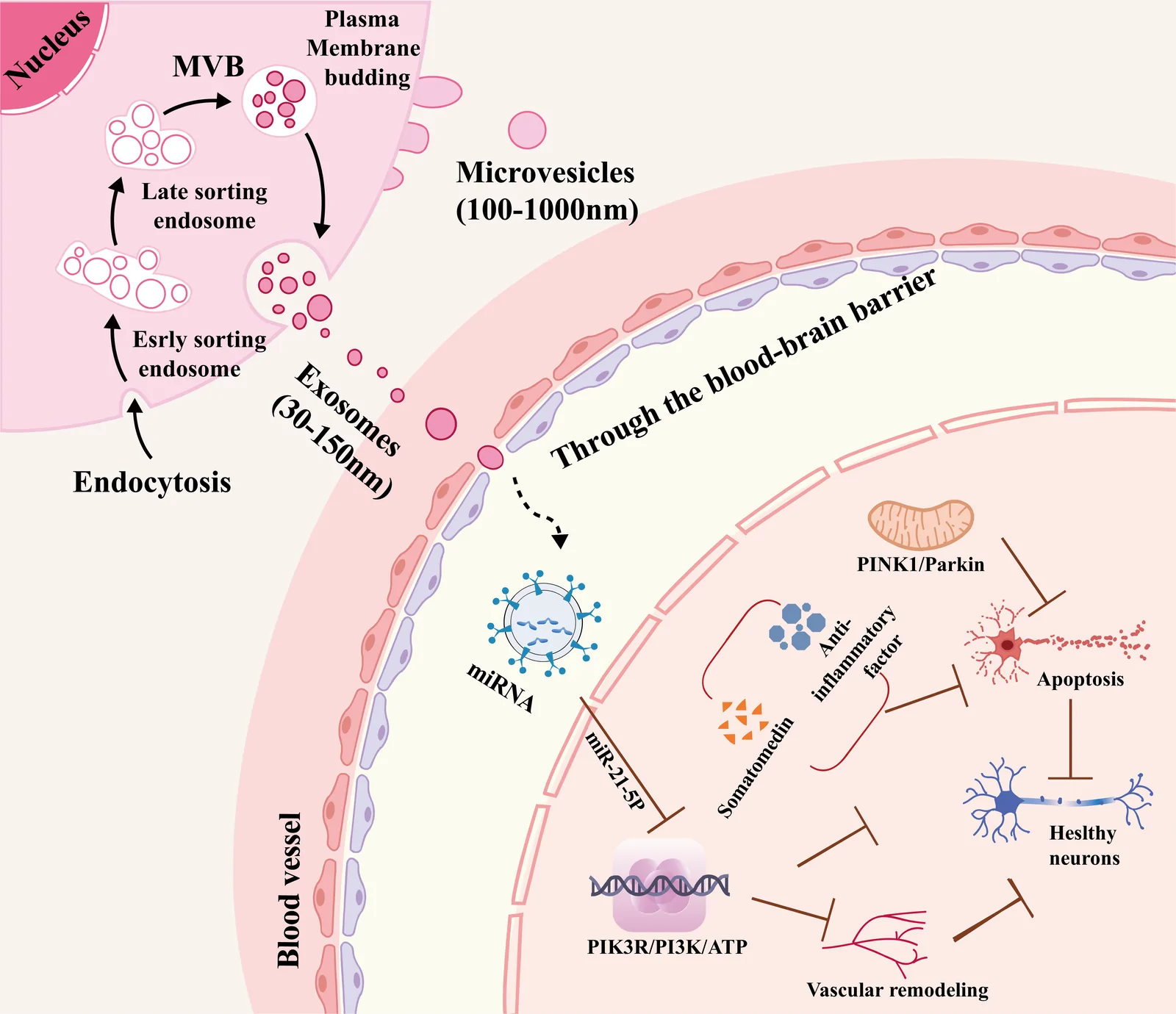
Molecular mechanisms of exosomes in CI.

**Table 1:** The interactions among microglia, astrocytes, oligodendrocytes, neurons, vascular cells, and immune cells.

**Cell Type**	**Interaction**	**Contribution to post-CI Pathogenesis**	**Contribution to Post-CI Self-healing**	**References**
Microglia	Interact with astrocytes, neurons, immune cells, modulate immune response.	Release pro-inflammatory cytokines, mediate cellular damage.	Remove necrotic tissue, secrete repair factors, modulate inflammatory homeostasis.	[13, 14, 31, 32]
Astrocytes	Intercommunicate with microglia, neurons and vascular cells, regulate inflammation and metabolism.	Form glial scar, barrier formation, local pro-inflammatory.	Secret neurotrophic factor, promote BBB repair and neuroprotection.	[15, 16]
Oligodendrocytes	Work closely with neurons and are influenced by astrocytes and microglia.	Myelin damage leads to impaired nerve conduction.	Remyelination restores nerve conduction and enhances potential for restoration of function.	[17, 18]
Neurons	Receive support from astrocytes and are influenced by microglia and immune cells.	Cell necrosis and apoptosis lead to disruption of neural networks.	Axonal regeneration, synaptic plasticity and remodeling, restoration of some neural functions.	[20-22]
Vascular cells	Work with astrocytes to repair the blood-brain barrier and regulate the infiltration of immune cells.	The destruction of the blood-brain barrier leads to the invasion of inflammatory cells, which exacerbates the damage.	Promote vascular neovascularisation and perfusion, and improve the local metabolic environment.	[28, 29]
Immune cells	Cross the BBB, interact with microglia and astrocytes, and regulate the local immune environment.	Exacerbating local inflammation and causing secondary damage to brain tissue.	Removing necrotic cells and promoting reparative inflammatory transformation.	[33, 34]

**Table 2:** Major cytokines and their roles in CI.

**Cytokine**	**Function**	**Mechanism**	**Research Progress**	**References**
TNF-α	Pro-inflammatory cytokines that exacerbate the inflammatory response after CI.	Activates the NF-κB signaling pathway and promotes the expression of apoptosis-related genes, while different effects (pro- or anti-apoptotic) can be induced *via* TNFR1 and TNFR2.	TNF-α inhibitors (*e.g*., Etanercept) show reduced inflammation and amelioration of CI injury; however, their complexity requires more precise targeting strategies.	[71, 72]
IL-1β	Pro-inflammatory factors that trigger an inflammatory response.	Activation of the inflammasome, which promotes the release of other pro-inflammatory factors, exacerbates inflammation and apoptosis after CI.	IL-1 receptor antagonists (IL-1RA) have shown reduction of infarct volume in animal models, and the potential of clinical studies (*e.g*., Anakinra) for ischemic stroke is being evaluated.	[73, 74]
IL-6	Dual action, both pro- and anti-inflammatory.	Regulation of the JAK/STAT signaling pathway *via* the gp130 receptor family is involved in vascular protection, neuronal survival and may also exacerbate chronic inflammatory responses.	Elevated early after CI, associated with neuroinflammation and brain damage; IL-6 blockers (*e.g*., Tocilizumab) have been studied to reduce inflammation-related brain damage.	[75-80]
IL-10	Anti-inflammatory factor, inhibits inflammation and protects brain tissue.	Inhibition of the NF-κB signaling pathway reduces the expression of pro-inflammatory cytokines (*e.g*., IL-1β, TNF-α) and promotes neuroprotection and tissue repair.	Reduction of cerebral infarct volume by overexpression of IL-10 was shown in animal experiments; its role in immunomodulation and protective inflammation is considered a potential therapeutic target.	[89, 90]

**Table 3:** Recent research on exosomes in CI.

**Source of Exosomes**	**Component**	**Function**	**Mechanism**	**Experiment Type**	**References**
Mesenchymal stem cells	-	Nerve regeneration	Promote cell survival, proliferation and differentiation.	*In vitro* and *in vivo*	[94]
Mesenchymal stem cells	-	Regulation of inflammation	Regulates neuroinflammation and promotes neuronal survival.	*In vitro* and *in vivo*	[95]
M2 Microglia	-	Regulating the function of neurons	Alleviates neuronal apoptosis after ischemic brain injury and promotes neuronal survival and functional recovery.	*In vivo*	[96]
Human umbilical cord mesenchymal stem cells	-	Possibly through its capabilities in nerve regeneration, remyelination, anti-inflammation, and anti-apoptosis	Promotion of nerve regeneration, remyelination, and synapse formation.	*In vivo*	[97]
Blood, cerebrospinal fluid, and urine	-	Regulation of inflammation	Regulate neuroinflammatory response and improve the survival environment of neurons.	*In vivo*	[98]
Adipose-derived stem cells	miR-21-5p	Regulation of inflammation	Alleviate the inflammatory response after CI through PIK3R1/PI3K/AKT signaling axis and provide neuroprotection after CI by promoting the polarization of M2 microglia.	*In vivo*	[109]
Neural stem cells	miR-150-3p	Neuroprotection	Inhibit CASP2 signaling pathway, thereby inhibiting neuronal apoptosis and promoting neuronal proliferation after brain injury.	*In vitro*	[110]
Astrocytes	miR-92b-3p	-	Attenuated neuron death and apoptosis.	*In vitro*	[110]
Astrocytes	Circular RNA circSHOC2	Regulate autophagy and miR-7670-3p/SIRT1	Suppress neuronal apoptosis.	*In vitro* and *in vivo*	[110]
Mesenchymal stem cells	-	Neuroprotection	Reduce cell death, and regulate related signaling pathways (such asPINK1/Parkin pathway), regeneration, and neural plasticity.	*In vivo*	[116, 117]
Plant	-	Nerve regeneration	Improve functional recovery by regulating the inflammatory response and promoting the differentiation of neural stem cells.	*In vivo*	[118, 119]
M2 Microglia	miR-124	Neuroprotection	Attenuates ischemic brain injury and promotes neuronal survival.	*In vitro* and *in vivo*	[121]

**Table 4:** Comparative analysis of technologies in studying CI.

**Technology**	**Functions**	**Advantages**	**Limitations**	**Applicable Scenarios**	**References**
Single cell sequencing	Analyze the genomic, transcriptomic, or epigenomic characteristics of individual cells to reveal cellular heterogeneity and dynamic changes.	High resolution, revealing molecular information at the single-cell level; It can analyze cell types and their functions in complex tissues or disease microenvironments.	High cost; high requirements for sample processing; large amount of data; complex analysis,	Cancer heterogeneity studies; Immune cell lineage analysis; Cellular function analysis in neurological diseases; Developmental biology research.	[143, 146, 147, 160]
Imaging technology	High resolution two-dimensional or three-dimensional imaging of tissues or cells; Cell morphology, distribution and interaction were observed.	Noninvasive, real-time observation; Suitable for the study of cell dynamics, structure and function; Some techniques can be combined with fluorescent labeling to achieve molecular specific imaging	Limited deep penetration (partial optical imaging); Sample preparation and labeling may affect the natural state; The equipment is expensive.	Intravital imaging studies; Organizational structure analysis; Neuronal connectivity studies; Dynamic observation of cerebral blood flow and brain injury.	[144, 162]
Nanotechnology	Accurately operate and study nanoscale substances; Design nanoscale materials for diagnosis, treatment or research.	High sensitivity; Multifunctional nanocarriers can be designed; It can improve targeting and reduce side effects when used for drug delivery; Strong versatility in multiple fields.	May cause biological toxicity; The *in vivo* behavior of some nanomaterials is unpredictable; High cost; Standardization issues.	Nanomedicine development; Molecular diagnostic markers; Treatment of cerebrovascular disorders (such as targeted delivery of drugs to treat cerebral ischemia); Biomarker detection.	[145, 154, 155]

**Table 5:** Summary of intercellular communication pathways in CI.

**Name**	**Communication Pathways**	**Molecular Basis**	**Functions**	**CI Effects**	**References**
Adhesion molecule	Cell surface molecules bind to neighboring cells or matrix and regulate adhesion and signal transduction.	Major molecules: ICAM-1, VCAM-1, *etc*. Receptors: integrins, calcineurin, *etc*.	Regulates intercellular adhesion and migration. Involved in the recruitment of inflammatory cells and homeostatic maintenance of the BBB.	ICAM-1 and VCAM-1 exacerbate inflammatory responses and tissue damage. Integrins promote angiogenesis and neuroprotection, as well as recruitment of inflammatory cells, exacerbating brain injury. Changes in calcineurin expression after CI may lead to disruption of the BBB, which aggravates brain injury. Cell adhesion promotes neural repair and regeneration.	[35-42]
Cytokine	Secreted outside the cell, spreads to neighboring or distal cells, activates signaling pathways by binding to specific receptors.	Common molecules: TNF-α, IL-1β, IL-6, IL-10, and others. Receptors: cell surface specific cytokine receptors.	Regulates inflammatory response, immune response, cell survival and apoptosis. Induces angiogenesis and participates in damage repair.	TNF-α and IL-1β promote inflammatory responses and exacerbate tissue damage, thereby exacerbating neuroinflammation and neuronal apoptosis. IL-10 is thought to play a role in inhibiting the inflammatory response and promoting microvascular repair.	[65, 66, 89, 90]
Exosomes	Released from the cell into the extracellular space by cytotoxicity and into the target cell by endocytosis.	Small vesicles encapsulated in lipid bilayers (30-150 nm).	Capable of delivering a wide range of biologically active molecules, including proteins, lipids and nucleic acids, regulating gene expression and signaling pathways. Regulates inflammation, angiogenesis, neuronal survival and regeneration.	MiRNAs in exosomes reduce inflammation and promote neuroprotection. It can promote cerebrovascular repair and tissue remodeling after ischemia by transmitting growth factors and signaling molecules.	[94, 95, 100, 110]
Gap junction	Direct transfer of ions, small molecules and signaling molecules through channels consisting of connexins.	Connexins: *e.g*., Cx43, Cx32. Structure: specialized tight junction channels between cell membranes.	Enables electrical and chemical signaling between cells. Coordinate synchronized activities in tissues and maintain intercellular homeostasis.	Gap junction opening under ischemic conditions may diffuse injury signals (*e.g*., Ca²^+^ overload, ROS). Aberrant expression of Cx43 affects neuronal survival and function.	[125, 126, 131, 135, 137, 138]

## LIST OF ABBREVIATIONS

ADSC-exosomes: Exosomes from Adipose-Derived Stem Cells
ASC-EVs: Adipose-Derived Stem Cell-Derived Extracellular Vesicles
ATP: Adenosine Triphosphate
BBB: Blood-Brain Barrier
BDNF: Brain-Derived Neurotrophic Factor
CASP2: Caspase 2
CEUS: Contrast-enhanced Ultrasound
CI: Cerebral Ischemia
Cx43: Connexin 43
ECs: Endothelial Cells
EVs: Extracellular Vehicles
GSH: Glutathione
HIF-1α: Hypoxia-Inducible Factor-1 Alpha
ICAM-1: Intercellular Adhesion Molecule-1
IL-10: Interleukin-10
IL-1β: Interleukin-1 Beta
IL-6: Interleukin-6
JAK/STAT: Janus Kinase/Signal Transducers and Activators of Transcription
JAK2/STAT3: Janus Kinase 2/Signal Transducer and Activator of Transcription 3
L1CAM: L1 Cell Adhesion Molecule
MAPK: Mitogen-Activated Protein Kinase
MRI: Magnetic Resonance Imaging
MSCs: Mesenchymal Stem Cells
MSI: Mass Spectrometry Imaging
mTOR: Mammalian Target of Rapamycin
NF-κB: Nuclear Factor Kappa-Light-Chain-Enhancer of Activated B Cells
NGF: Nerve Growth Factor
PCs: Pericytes
PDGF: Platelet-Derived Growth Factor
PI3K/Akt: Phosphatidylinositol 3-Kinase/Protein Kinase B
PIK3R1/PI3K/AKT: Phosphatidylinositol-3-kinase Regulatory Subunit 1/Phosphatidylinositol 3-kinase/Protein Kinase B
PINK1/Parkin: PTEN-induced Putative Kinase 1/Parkin
RhoA: Ras Homologous Gene Family Member A
scRNA-seq: Single-cell RNA sequencing technology
SIRT1: Silent Mating Type Information Regulation 2 Homolog 1
TGF-β: Transforming Growth Factor Beta
TNF-α: Tumor Necrosis Factor Alpha
VCAM-1: Vascular Cell Adhesion Molecule-1
VEGF: Vascular Endothelial Growth Factor
